# Sequence similarity between the erythrocyte binding domain of the *Plasmodium vivax *Duffy binding protein and the V3 loop of HIV-1 strain MN reveals a functional heparin binding motif involved in binding to the Duffy antigen receptor for chemokines

**DOI:** 10.1186/1743-422X-8-523

**Published:** 2011-11-28

**Authors:** Michael J Bolton, Robert F Garry

**Affiliations:** 1 Department of Microbiology and Immunology, Tulane University, 1430 Tulane Avenue, New Orleans, LA 70112, USA; 2 Vaccine and Infectious Disease Institute, Fred Hutchinson Cancer Research Center, Division of Allergy and Infectious Diseases, University of Washington, 1100 Fairview Avenue, Seattle, WA 98109, USA

## Abstract

**Background:**

The HIV surface glycoprotein gp120 (SU, gp120) and the *Plasmodium vivax *Duffy binding protein (PvDBP) bind to chemokine receptors during infection and have a site of amino acid sequence similarity in their binding domains that often includes a heparin binding motif (HBM). Infection by either pathogen has been found to be inhibited by polyanions.

**Results:**

Specific polyanions that inhibit HIV infection and bind to the V3 loop of X4 strains also inhibited DBP-mediated infection of erythrocytes and DBP binding to the Duffy Antigen Receptor for Chemokines (DARC). A peptide including the HBM of PvDBP had similar affinity for heparin as RANTES and V3 loop peptides, and could be specifically inhibited from heparin binding by the same polyanions that inhibit DBP binding to DARC. However, some V3 peptides can competitively inhibit RANTES binding to heparin, but not the PvDBP HBM peptide. Three other members of the DBP family have an HBM sequence that is necessary for erythrocyte binding, however only the protein which binds to DARC, the *P. knowlesi *alpha protein, is inhibited by heparin from binding to erythrocytes. Heparitinase digestion does not affect the binding of DBP to erythrocytes.

**Conclusion:**

The HBMs of DBPs that bind to DARC have similar heparin binding affinities as some V3 loop peptides and chemokines, are responsible for specific sulfated polysaccharide inhibition of parasite binding and invasion of red blood cells, and are more likely to bind to negative charges on the receptor than cell surface glycosaminoglycans.

## Introduction

The human immunodeficiency virus type 1 (HIV-1), the human malaria, *Plasmodium vivax*, and the monkey malaria, *P. knowlesi*, have ligands that bind to chemokine receptors and mediate cell invasion. The surface glycoprotein gp120 (SU) of HIV-1 binds to CCR5 and CXCR4 as the major coreceptors for infecting CD4+ T-lymphocytes in vivo, and changes in the amino acid sequence of the V3 loop of gp120 can change viral tropism from CCR5 using (R5) to CXCR4 using (X4) to both (R5X4) [1-4]. The V3 loop region of gp120 also provides a neutralizing epitope, and can bind glycosaminoglycans and other polyanions which inhibit viral infection [5-14].

*P. vivax *uses a Duffy binding protein (PvDBP) to bind the Duffy antigen receptor for chemokines (DARC) and invade human reticulocytes. *P. knowlesi *has three proteins, the *P. knowlesi α*, *β*, and γ proteins which can mediate binding to rhesus erythrocytes, and the *P. knowlesi α *protein (PkDBP) can bind to human and rhesus DARC. PvDBP, PkDBP, *P. knowlesi β*, and *γ *proteins are members of a Duffy Binding Ligand (DBL) family of erythrocyte binding proteins with conserved regions of homology which bind to many receptors. Region II within the family, as defined by conserved cysteine residues, is responsible for erythrocyte binding, and region II of PkDBP has been shown to be inhibited by glycosaminoglycan binding [15].

In a separate report, we describe an amino acid sequence similarity between subdomain 1 in DBP region II and the V3 loop of HIV strain MN [16]. Within subdomain 1 and this V3 loop are consensus BBXB heparin binding motifs (HBM), where B is a basic amino acid and X is any amino acid. This HBM is conserved in many DBL family members, and we previously found that alanine substitutions at this site in PvDBP and PkDBP abrogated DARC binding. RANTES is a natural ligand of both CCR5 and DARC and can inhibit both HIV and DBP binding to their respective receptors. SDF-1 is a natural ligand for CXCR4, and both RANTES and SDF-1 have HBM and are known to bind sulfated polysaccharides [17].

One possible function of the HBM in chemokines, HIV and DBPs is to associate with cell surface proteoglycans. Alternatively, HBMs could participate in binding to negatively charged amino acid side chains on the chemokine receptors. RANTES is known to bind to sulfated polysaccharides as part of its processing and function, but tyrosine sulfation of CCR5 is also important for binding of chemokines and HIV, and sulfation of Tyr 41 on DARC is important for DBP binding. Here we designed a peptide from PvDBP subdomain 1 that contains the HBM, tested its ability to bind sulfated polysaccharides, and compared it to the binding of the PvDBP, PkDBP, *P. knowlesi β *and *γ *proteins, HIV V3 loop peptides and RANTES to see if they shared similar binding specificities.

## Materials and methods

### Polyanions

Ca-spirulan, Na-spirulan, and Na-hornan (Na-HOR) were kindly provided by Toshimutsu Hayashi, Department of Virology, Toyama Medical and Pharmaceutical University, Sugitani, Toyama, Japan [18,19]. Heparin, dextran sulfate, and pentosan polysulfide were obtained from Sigma-Aldrich (St. Louis, MO).

### Peptide preparation

**Figure 1:**
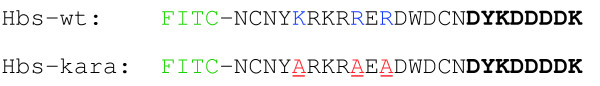
**Design of peptides hbs-wt and hbs-kara**. The consensus heparin binding motif in the DBP V3-like peptide of PvRII is contained in hbs-wt. The same alanine substitutions from a nonbinding mutant of PvRII (pv22KARA), tested in a previous study, were included in hbs-kara [16]. Alanine-substituted amino acids are in blue and red. The N-terminus is to the left and is FITC-conjugated. The final residues at the C-terminus, DYKDDDDK, represent the FLAG epitope and are in bold.

Peptides based on the wild type (wt) putative polyamine binding site of the PvDBP and a non-binding mutant, pvR22KARA (Figure 1) were obtained from Gene med Synthesis, Inc. (San Francisco, CA). The synthesis included N-terminal fluoresce in conjugation and HULK purification to greater than 80%. Peptides of the V3 loop were obtained from the NHI AIDS Reagent Program (NHI AIDS Reagent Program, Rockville, Md.)

### Heparin-sepharose columns

The binding affinity of the PvDBP HBM and V3 loop peptides for heparin was determined by chromatography on a heparin-Sepharose column. Heparin-Sepharose CL-6B beads (Pharmacia Biotech) were swollen in 50 mM Tris-HCl pH 7.5 (column buffer), degassed for 1 h, and 1 ml of slurry was added to a 10 ml column. The column was equilibrated with 10 volumes of column buffer. Peptides were added at 1 mg/ml in 300 μl and allowed to enter the column. The column was washed with 3 ml of column buffer. The peptide was eluted with 3 ml volumes of increasing NaCl concentrations of 0.01, 0.15, 0.5, 1.0 and 2.0 M, and 0.5 ml fractions were collected. The column was regenerated between peptides by adding alternating 3 ml volumes of 0.1 M Tris-HCl, 0.5 M NaCl, pH 8.5 and 0.1 M NaOAc, 0.5 M NaCl, pH 5.0 for three cycles. The column was re-equilibrated with 10 vol. of column buffer before adding the next peptide. Fractions were measured for absorbance at 280 nm on a spectrophotometer.

### *P. Knowlesi *in vitro culture

Whole blood from rhesus macaques was collected in 10% CPD and allowed to separate overnight at 4°C. The erythrocyte phase was washed in RPMI with L-glutamine and supplemented with 25 mM HEPES, 300 μM hypoxanthine, 10 μM thymidine, 1.0 mM sodium pyruvate, and 11 mM glucose. This RPMI with malaria supplements was then used to prepare malaria culture medium by adding to a final concentration of 0.24% sodium bicarbonate and 0.2% Albumax-I (Life Tech, Gibco BRL). Cultures were maintained at a hematocrit of 10% in malaria culture medium under an atmosphere of 5% O_2_, 5% CO_2_, balanced N_2 _(Air Liquide, Houston, TX) at 38°C.

### Erythrocytes

Blood was collected in 10% citrate phosphate dextrose (CPD) and stored at 4°C unwashed for up to 4 weeks, or washed in RPMI with malaria supplements and stored in malaria culture medium at 50% hematocrit for up to 2 weeks. The DARC+ human erythrocytes used in the erythrocyte binding assay and the *P. knowlesi *erythrocyte invasion assay had the phenotype Fy(a^-^b^+^) as determined by standard blood banking methods using anti-Fya and anti-Fyb antisera (Gamma Biologicals, Houston, TX). Erythrocytes were washed three times in DMEM (Gibco BRL) and resuspended to a hematocrit of 10% in complete DMEM for the erythrocyte binding assay. Erythrocytes used in the *P. knowlesi *erythrocyte invasion assay were washed three times and resuspended to a hematocrit of 10% using malaria complete RPMI.

### Percoll purification of schizont-infected erythrocytes

Cultures of *P. knowlesi *at 5-10% infected erythrocytes were washed three times in RPMI with malaria supplements and 10% FBS and brought up to a hematocrit of 10%. A 50% Percoll solution was made by adding 0.45 vol 1× PBS, 0.05 vol 10× PBS and 0.5 vol Percoll (Sigma). Two ml of the washed culture was overlaid on 2 ml of the 50% Percoll solution in a 4 ml polystyrene tube and centrifuged for 20 min at 2100 RPM in a Sorvall centrifuge. The ring of cells at the interface was removed, pooled and washed three time in 1× PBS. The pellet was brought up in malaria culture medium to 2 × 10^7 ^cells/ml.

### *P. Knowlesi *erythrocyte invasion assay

Human Duffy Fy(a^-^b^+^) erythrocytes were washed in complete malaria medium and 2 × 10^7 ^washed cells were added to increasing concentrations of sulfated polysaccharide in malaria culture medium at final volume of 900 μl for 1 h at room temperature. To each tube of sulfate polysaccharide-treated erythrocytes, 100 μl or 2 × 10^6 ^schizont-infected erythrocytes was added and placed in a well of a polystyrene 24-well plate (Becton-Dickinson). The cultures were maintained under a blood-gas atmosphere at 38°C for 8 h to allow the infected erythrocytes to rupture and release free merozoites capable of infecting new erythrocytes and developing to ring-stage trophozoites. The culture was centrifuged at 2100 RPM for 3 min and a thin smear was made from the pellet. The thin smear was fixed with methanol and stained with Leukostat Solution B (100 mg Eosin Y+ 300 μl 37% formaldehyde +400 mg sodium phosphate dibasic +500 mg potassium phosphate monobasic, q.s. to 100 ml with dH_2_O), rinsed, and stained with Leukostat Solution C (47 mg Methylene Blue +44 mpp Azure A +400 mg sodium phosphate dibasic +500 mg potassium phosphate monobasic, q.s to 100 ml with dH_2_O). The percentage of erythrocytes infected with ring-stage trophozoites per 2000 erythrocytes was determined at 1000×. Percentage inhibition of invastion was determined by dividing the percentage of ring-stage parasites at each polyanion concentration by the percentage of ring-stage parasites at 0 μg/ml of the polyanion, multiplying by 100 and subtracting this value from 100 [20]

### PvRII erythrocyte binding assay

COS-7 cells were transfected by Lipofectamine with 1-2 μg of pHVDR22 DNA, a plasmid kindly provided by L. Miller which expresses region II of the DBP of *P. vivax *on the cell surface as a chimera with the HSV gD protein [21]. Duffy Fy (a-b+) erythrocytes were washed three times in RPMI 1640, resuspended to a hematocrit of 1% in 1 ml of complete DMEM with polyanions at concentrations of 0, 1, 10, 100, and 1000 μg/ml. This suspension was swirled over aspirated COS-7 cells 40-60 h after transfection and allowed to settle over 2 h at 37°C. The COS-7 cells were then washed three times with 2 ml of PBS to remove nonadherent erythrocytes. The number of adherent erythrocyte rosettes was scored in 20 randomly chosen fields at a magnification of 40 using an inverted microscope. Percentage inhibition of binding was determined by dividing the number of rosettes at each polyanion concentration by the percentage of rosettes at 0 μg/ml of the polyanion, multiplying by 100 and subtracting this value from 100.

### PvDBP peptide BSA-heparin ELISA

Polyvinyl chloride 96-well microtiter plates were coated with 5 μg/ml heparin-albumin (Sigma) in a volume of 100 μl per well in 50 mM Tris-HCl pH 7.5 wash buffer overnight at room temperature. Plates were washed three times with wash buffer and blocked for 2 h at room temperature with 1% BSA in wash buffer at 400 ul per well. For polyanion blocking experiments, WT or mutant DBP polyanion binding site peptides were diluted to 10 μg/ml in a final concentration of polyanions at 0, 0.1, 1, 10, 100, or 1,000 μg/ml in wash buffer and added at 100 μl per well for 2 h. For controls, the DBP peptides were added at 10 μg/ml in 0.01, 0.15, 0.5, 1.0, and 2.0 M NaCl 50 mM Tris HCl pH 7.5 (data not shown). Plates were washed three times with wash buffer. Chicken anti-DYKDDDDK epitope antibody (Aves Labs, OR) at 1:5000 in 1% BSA wash buffer was added at 100 μl per well for 1 h at room temperature. Rabbit anti-chicken horseradish peroxidase antibody (Jackson ImmunoResearch) was added at 1:5000 in 1% BSA wash buffer at 100 μl per well for 1 h at room temperature. Plates were washed three times with wash buffer. The reaction was developed with 100 μl per well of 2% 3,3',5,5'-tetramethylbenzidine 0.1 M NaOAc containing 0.001% hydrogen peroxide for about 5 min. The reaction was stopped with 100 μl per well of 1 M phosphoric acid. Absorbance measurements were made at 450 nm on a Biotek 133 microtiter plate reader. Percentage inhibition of binding was determined by dividing the absorbance at each polyanion concentration by the absorbance at 0 μg/ml of the polyanion, multiplying by 100 and subtracting this value from 100.

### RANTES BSA-heparin ELISA

The same ELISA format used for the PvDBP Peptide-BSA-heparin ELISA described above was used for a competitive ELISA to detect RANTES binding to heparin, and competitors to this binding. Wells were coated with BSA-heparin, blocked with BSA and washed. A volume of 100 μl of 5 nM RANTES in wash buffer supplemented with 0, 0.5, 5, 50, 500 or 5000 nM peptide was added to triplicate wells. After incubation for 1.5 h at room temperature, the plates were washed in wash buffer and 100 μl of biotinylated anti-RANTES monoclonal antibody (R&D Systems) was added at 1:500 in 0.1% BSA wash buffer. After 1 h at room temperature, the plates were washed and 100 μl of streptavidin-horseradish peroxidase (Jackson ImmunoResearch) was added at 1:2000 in 0.1% BSA wash buffer. After 1 h at room temperature, the plates were washed. The reaction was developed as above. Percentage inhibition of binding was determined by dividing the absorbance at each peptide concentration by the absorbance at 0 nM of the peptide, multiplying by 100 and subtracting this value from 100.

### Heparitinase digestion

Red blood cells were washed 3 times in PBS and resuspended at a hematocrit of 10%. For the PvRII erythrocyte binding assay, 1 ml of 10% hct blood was used. To each 1 ml of red blood cells, 0, 0.001, 0.002 or 0.01 International Units (which correspond to 0.6, 1.2 and 6 Sigma Units, respectively) of Heparitinase (EC 4.2.2.8, Seikagaku America, Falmouth, MA) was added. The cells were incubated at 43°C for 90 min. with intermittent agitation. An aliquot of 30 μl of the cells was taken for analysis by flow cytometry (data not shown). The remaining cells were then centrifuged at 3000 RPM for 5 min., and resuspended in 1 ml of complete for use in the PvRII erythrocyte binding assay.

## Results

### Region II of the *P. Vivax *DBP is blocked from binding to DARC by the same polyanions that inhibit X4 HIV strains

**Figure 2:**
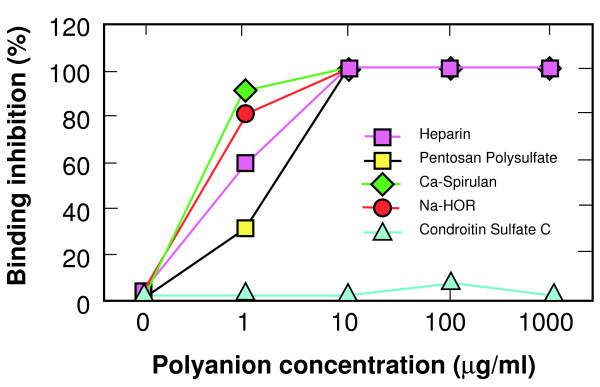
**Polyanion inhibition of PvRII binding to DARC+ erythrocytes**. Heparin, pentosan polysulfate, and the algal-derived sulfated polysaccharides Ca-spirulan, and Na-hornan (Na-HOR) have been shown to have potent inhibitory activity against HIV binding and infection. Chondroitin sulfate C does not and serves as a control. Inhibition of DARC+ erythrocytes binding to the DBP binding site was determined by comparing the number of COS-7 cells expressing pvRII with rosettes of polyanion-treated DARC+ human erythrocytes (per 20 fields at 200× magnification) with the number of rosettes of untreated erythrocytes.

**Figure 3:**
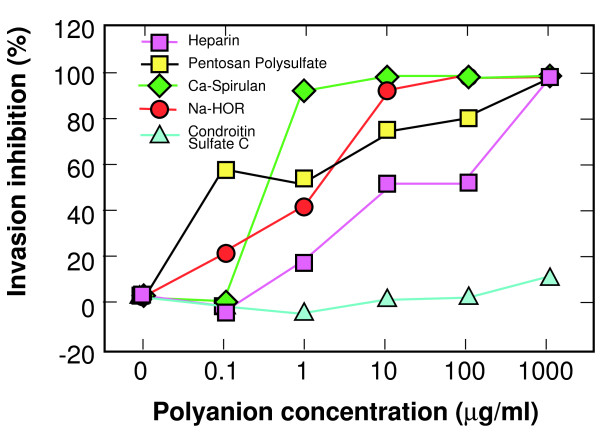
**Polyanion inhibition of *P. knowlesi *invasion of DARC+ erythrocytes**. The same polyanions used to inhibit the pvRII binding assay shown in Figure 2 were used in the *P. knowlesi *invasion assay. The inhibition of invasion was determined by subtracting the number of chemokine-treated DARC+ human erythrocytes invaded by *P. knowlesi *merozoites (per 2000 erythrocytes) from the number of untreated DARC+ human erythrocytes invaded by *P. knowlesi *merozoites, and dividing by the number of untreated, invaded erythrocytes.

Within the DBP V3-like peptide is a site that conforms to the consensus heparin binding sequences BBXB and BBBXXB, where B represents a basic amino acid and X represents any amino acid including basic amino acids. Some strains of HIV, such as MN, contain a consensus heparin binding motif in the V3 loop, and many X4 strains can be inhibited from infecting target cells by polyanions which may bind to the V3 loop. Polyanions that have been shown to inhibit HIV infection include pentosan polysulfate, heparin, and the algal-derived sulfated polysaccharides Na-spirulan, Ca-spirulan and Na-hornan (Na-HOR) [18,22]. These polyanions also inhibited the binding of DARC+ erythrocytes to PvRII in a dose-dependent manner (Figure 2). They also block *P. knowlesi *invasion of DARC+ erythrocytes (Figure 3). Chondroitin sulfate C represents a polyanion with similar charge to heparin, but differs in the conformational placement of those charges. Chondroitin sulfate C does not block PvRII binding to DARC, or *P. knowlesi *invasion of DARC+ erythrocytes, suggesting that the interaction between PvRII and polyanions is related to conformation as well as charge. The same is true for inhibition of the V3 loop by polyanions [5,7-12,14,23-25].

### A peptide based on the consensus heparin binding motif in the DBP V3-like peptide binds to heparin with the same affinity as V3 loop peptides of X4 HIV strains and recapitulates polyanion inhibition of PvRII binding to DARC

A peptide based on the consensus heparin binding motif in the DBP V3-like peptide was designed to test the affinity of this site for heparin and compare it to V3 loop peptides and RANTES. Based on results with an alanine substitution mutant of the consensus heparin binding motif in the DBP V3-like peptide, two peptides were designed; one contains the wild type heparin binding site (hbs-wt), and the other contains the same alanine substitutions as the pv22KARA construct (hbs-kara) found in our previous work to abrogate binding to DARC [16]. The peptides are FITC conjugated at the N-terminus and terminate at the carboxyl end with the DYKDDDDK "flag epitope" sequence for fluorescence or antigenic detection, respectively. They are identical with the exception of the alanine substitutions (Figure 1).

**Figure 4:**
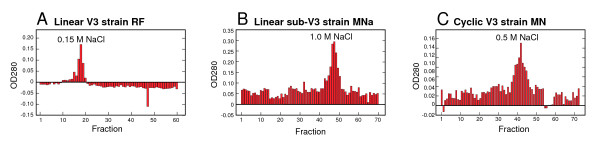
**Heparin-Sepharose column fractions**. Peptides in Table 1 were bound to a heparin-Sepharose column and eluted with NaCl at concentrations of 0.01, 0.15, 0.5, 1.0, and 2.0 M. The optical densities of the fractions of most peptides were determined in the at a wavelength of 280 nm. Representative examples of the optical densities of fractions from three peptides in Table 1 are shown in panels A-C in which the NaCl concentration at which the peak fraction eluted is written above the peak.

The hbs-wt, hbs-kara, an assortment of V3 loop peptides, and RANTES were bound to a heparin-Sepharose column and eluted with 0.01, 0.15, 0.5, 1.0 or 2.0 M NaCl. The NaCl concentration required to elute the peptides provides a relative value for the affinity between the peptide and heparin, and is directly proportional to the K_d _value. The 0.15 M NaCl concentration is physiologically relevant but reflects weak binding, 0.5 M indicates moderate binding, and 1.0 M and above represents strong binding (reviewed in chapter 6 of "Heparin-Binding Proteins" [26]. Most of the peptides could be detected in the fractions by their absorbance of light at 280 nm on a spectrophotometer, with the exception of the linear V3 loop peptide of strain IIIB that lacks aromatic side chains. The Pierce BCA Protein Assay was used to detect this peptide (Figure 4).

The hbs-wt peptide eluted at 0.5 M NaCl (Table 1). The hbs-kara peptide did not bind to the column at all, eluting with the wash buffer. In a subsequent experiment, without the wash step, it eluted with 0.01 M NaCl. The cyclic V3 loop peptide of X4 HIV strain MN also eluted at 0.5 M as did the linear peptide of X4 strain IIIB. Of the linear peptides that are overlapping subunits of the MN cyclic peptide, the peptide containing the consensus heparin binding motif eluted at 1.0 M. This is a stronger interaction than the cyclic full-length V3 peptide. Other V3 peptides based on consensus sequences of subtypes B and EA, or specific strains RF and SF2, eluted at 0.15 M. RANTES eluted at 0.5 M. There was no direct relationship between net charge of the peptides and affinity to heparin, as the cyclic MN peptide with a charge of +7 eluted at a lower NaCl concentration than the linear peptide at a charge of +6. The most neutral net charge in the tested group of peptides was 0 for the hbs-wt, and it eluted at 0.5 M NaCl.

**Table 1:** Heparin-Sepharose peak fractions of peptides of the V3 loop of HIV, hbs-wt and hbs-kara of the *P. vivax *DBP, and RANTES

Peptide	Amino Acid Sequence	Charge @ pH 7.0	[NaCl] of Peak Fraction
Cyclic V3 strain MN	CT**R**PNYN**KRKR**IDIGPG**R**AFTT**K**NIIGTI**R**QAHC	+7	0.5 M

Linear sub-V3 strain MNa	CT**R**PNYN**KRKR**IHIPGPG**R**A	+6	1.0 M

Linear sub-V3 strain MNb	**R**IHIGPG**R**AF YTT**K**NIIGTI	+3	0.15 M

Linear sub-V3 strain MNc	YTT**K**NIIPTI**R**QAHCNIS**R**A	+3	0.15 M

Cyclic V3 subtype EA	CTSITIGPGQVFY**R**TGC	+1	0.15 M

Cyclic V3 Subtype B	C**K**GI**R**IPGP**R**AVYAAEC	+2	0.15 M

Linear V3 Strain RF	**K**SIT**K**GPG**R**VIYATG	+3	0.15 M

Linear V3 Strain SF2	**K**SIFIGPG**R**AFHTTG	+2	0.15 M

Linear V3 Strain IIIB	T**R**PNNNT**RK**SI**R**IQ**R**GPG**R**AFVTIG**K**IGNM**R**	+8	0.5 M

Hbs-wt	NCNY**KRKRR**E**R**DWDCNDY**K**DDDD**K**	0	0.5 M

Hbs-kara	NCNYA**RK**A**R**EADWDCNDY**K**DDDD**K**	**-**3	0.01 M

RANTES	M**K**VSAA**R**LAVILIATACAPASASPYSSDTTP		

	CCFAYIA**R**PLP**R**AHI**K**EYFYTSG**K**CSNPAVV		

	FVT**RK**N**R**QVCANPE**KK**WV**R**EYINSLEMS	+7	0.5 M

**Figure 5:**
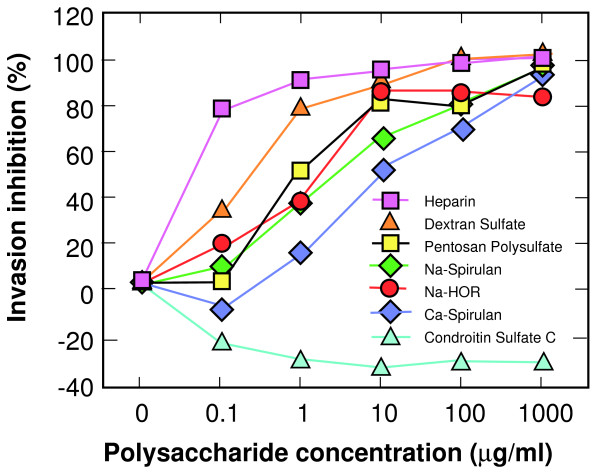
**Polyanion inhibition of the hbs-wt peptide binding to heparin in the BSA-heparin ELISA**. An ELISA was used to determine the inhibition of the hbs-wt peptide binding to heparin by various polyanions. The hbs-wt peptide was added to BSA-heparin coated plates and detected by a horse-radish peroxidase conjugated antibody that recognizes the FLAG epitope. Polyanions used to inhibit hbs-wt peptide binding to heparin included those used in the pvRII binding and *P. knowlesi *invasion assays. Also included were dextran sulfate and sodium spirulan which have also been shown to be potent inhibitors of X4 HIV strains. Percentage inhibition of binding was determined by dividing the absorbance at each polyanion concentration by the absorbance at 0 μg/ml of the polyanion.

An ELISA based on coating plates with BSA-heparin and determining the binding of the hbs-wt peptide, or hbs-kara as a control, was developed. This assay showed high sensitivity for detecting bound hbs-wt peptide with low background as demonstrated by low signal produced by the hbs-kara control (data not shown). The binding was inhibited at the same NaCl concentrations that eluted the hbs-wt peptide in the heparin-Sepharose column at 0.5 M and above (data not shown). When sulfated polysaccharides were added to the ELISA, they inhibited the binding of the hbs-wt peptide in a dose-dependent manner. The same sulfated polysaccharides found to be inhibitory in the erythrocyte invasion assay and the PvRII region binding assay where inhibitory in the ELISA, with no inhibition from chondroitin sulfate C. (Figure 5).

### V3 loop peptides from HIV-1 strain MN have different polyanion binding specificities than the DBP subdomain 1 HBM peptide

**Figure 6:**
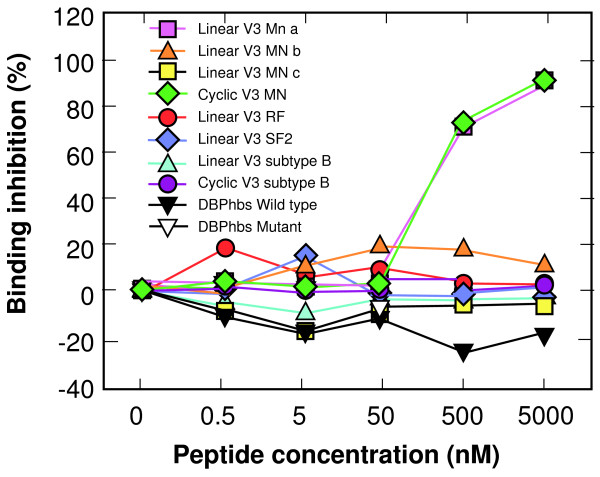
**Competitive Inhibition of RANTES-ELISA with V3 loop and hbs-wt peptides**. An ELISA format similar to that in Figure 5 was used to measure RANTES binding to heparin and compare the ability of various peptides to compete with this binding. A fixed concentration of 5 nM RANTES was mixed with various concentrations of competitors before addition to BSA-heparin coated plates. RANTES was then detected by an anti-RANTES mAb, a biotinylated secondary antibody and streptavidin conjugated to horseradish peroxidase. Percentage inhibition of binding was determined by dividing the absorbance at each peptide concentration by the absorbance at 0 nM of the peptide.

The heparin affinities of hbs-wt peptide, the cyclic V3 loop peptide of HIV-1 strain MN, and RANTES were the same when tested in heparin-Sepharose columns, and X4 strains of HIV, PvRII and hbs-wt can be inhibited by the same polyanions, but not chondroitin sulfate. To see if there are differences in the specificity by which the V3 peptides and the hbs-wt peptide bind to heparin, an ELISA based on RANTES binding to BSA-heparin was developed much like the hbs-wt ELISA. The only difference to the hbs-wt ELISA is that RANTES is substituted for the hbs peptide, and detection is through a biotinylated mouse anti-RANTES monoclonal antibody followed by horseradish peroxidase-conjugated streptavidin. The V3 loop peptides and hbs peptides were added at different concentrations to compete with RANTES at a fixed 5 nM. The linear and cyclic V3 loop peptides of strain MN that had significant binding on heparin-Sepharose were able to compete with RANTES binding to BSA-heparin in a dose-dependent manner, but the hbs-wt peptide was not (Figure 6).

### The subdomain 1 HBM has a conserved role in the DBP protein family for binding to diverse receptors, but only members of the family that bind to DARC are inhibited by polyanions

**Figure 7:**
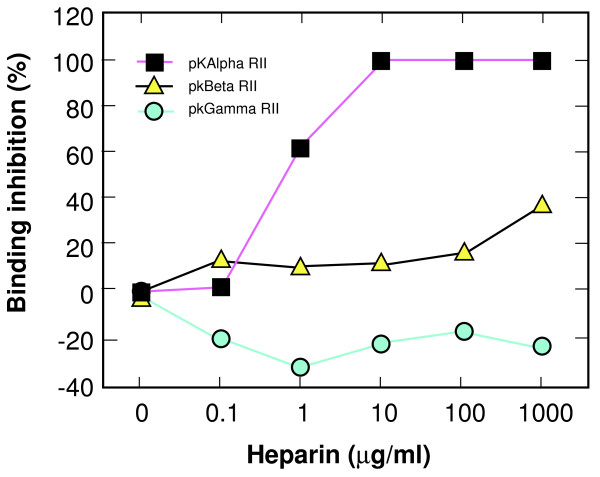
**Heparin inhibition of region II of *P. knowlesi *EBPs binding to rhesus erythrocytes**. The *P. knowlesi *alpha, beta and gamma proteins are members of the DBP family, bind rhesus erythrocytes by region II, and contain a consensus HBM. Only the alpha protein binds DARC. The PvRII binding assay shown in Figure 2 was repeated using plasmids to express the *P. knowlesi *EBPs, and using rhesus erythrocytes. Inhibition was determined in the same manner as the PvRII binding assay.

Studies by Ranjan and Chitnis have identified a site in PvRII in the C-terminal flanking region to the DBP V3-like peptide, between C4 and C7, that contain residues necessary for DARC binding [27]. This study also showed that the C1-C4 region of the *P. knowlesi *beta protein, a member of the DBP family that does not bind to DARC, was capable of substituting for the *P. vivax *C1-C4. Upon closer inspection, the consensus heparin binding motif is well conserved in the DBP family, with great similarity between proteins that bind different receptors [16]. The *P. knowlesi *alpha and gamma proteins have an identical consensus heparin binding site, but only alpha binds to DARC. To see if the consensus heparin binding motif may play a similar role in the binding proteins of other members of the DBP family, the same three alanine substitutions found in pv22KARA were introduced in our previous report by site directed mutagenesis into the plasmids pHKADR22, pHKBDR22, and pHKGDR22. This yielded the constructs pkalphaKARA, pkbetaKARA, and pkgammaKARA, which contain the K221, R224, and R227 alanine substitution in the *P. knowlesi *alpha, beta, and gamma genes, respectively. All three of these mutants failed to bind rhesus erythrocytes when expressed in COS-7 cells, whereas the parent vectors bind very well [16]. When binding of rhesus erythrocytes to the wild type plasmids in COS-7 cells was measured in the presence of polyanions, only the DARC binding alpha protein was inhibited in a dose-dependent manner (Figure 7).

### Heparitinase digestion of erythrocytes does not inhibit PvRII binding to DARC or *P. Knowlesi *invasion of DARC+ human erythrocytes

**Figure 8:**
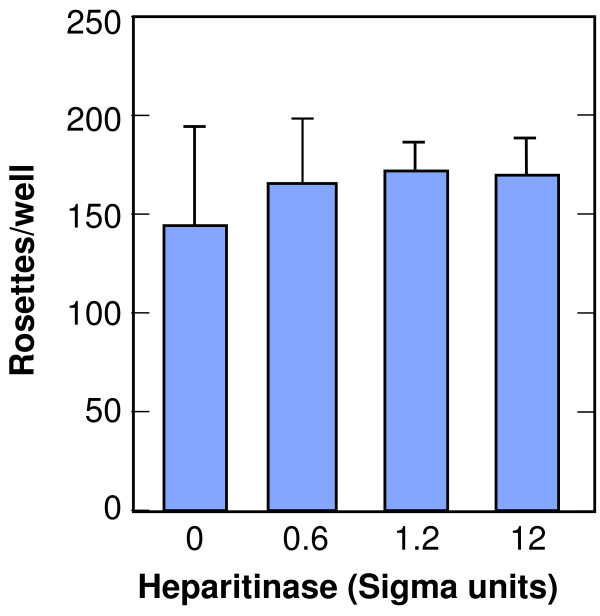
**PvRII binding to heparitinase treated DARC+ erythrocytes**. DARC+ human erythrocytes were digested with heparitinase I, an endoglycosidase specific for heparan sulfate. The number of rosettes of heparitinase-treated erythrocytes on COS-7 cells transfected with the pvRII expression vector, pHVDR22, is shown as the mean of three separate treatments.

To determine if polyanion binding and inhibition of PvRII was an indication that heparan sulfate on the surface of RBC was required for DARC binding, erythrocytes were digested with heparitinase, which specifically cleaves heparan sulfate. There was no significant difference in the binding or invasion of cells treated with increasing concentrations of heparitinase (Figure 8).

## Discussion

Members of the Duffy Binding Protein family have been found to bind to sulfated polysaccharides in a manner that inhibits their attachment to a variety of cell surface receptors. HIV can also be inhibited from chemokine receptor binding by sulfated polysaccharides, a property that has been the impetus for testing many microbicides in clinical trials. In a previous study, we examined similarities between the V3 loop of HIV-1 strain MN and the subdomain 1 of the Duffy Binding Protein of *P. vivax *that includes an HBM. This HBM is necessary for DARC binding and is conserved in the DBP family[16]. RANTES is a natural ligand of both the HIV coreceptor CCR5 and DARC, blocks binding of HIV and DBPs to these receptors, respectively, and contains an HBM. SDF-1 is the natural ligand for the HIV coreceptor CXCR-4 and contains a HBM. RANTES and SDF-1 bind to proteoglycans as part of their function, but also require tyrosine sulfation of CCR5 and CXCR-4 for binding, and their HBM that may function in both roles [28,29].

We hypothesize that the DBP and SU have convergently evolved to use chemokine receptors for binding, and may mimic the GAG and/or sulfated tyrosine binding functions of the chemokines using the V3 loop of SU and the HBM of subdomain 1 of the DBP. Here, we compared the binding specificity and affinity of HIV V3 loops, DBPs, a peptide containing the DBP HBM, and RANTES for binding to sulfated polysaccharides to assess whether they shared common binding mechanisms to chemokine receptors. In our first set of experiments we showed that the same sulfated polysaccharides that inhibit HIV binding and infection can also inhibit *P. knowlesi *infection of human erythrocytes, which is mediated by the *P. knowlesi *DBP binding to DARC. Using a previously described binding assay, we found the same sulfated polysaccharides inhibit *P. vivax *DBP region II from binding to DARC. In each of these and subsequent experiments, chondroin sulfate C was used as a negative control showing that not only the negative charges of the polysaccharides, but their specific orientation are important for inhibition.

To see if subdomain 1 of region II of PvDBP, which contains the HBM, may be involved in sulfated polysaccharide inhibition of DARC binding, we explored the affinity and specificity of a peptide based on this site for binding to sulfated polysaccharides. We found that a peptide containing the putative HBM (hbs-wt) does indeed bind to heparin, with a physiologically significant affinity similar to that of RANTES. In a previous study, we substituted alanine at K221, R224, and R227 of the PvDBP region II, which replaced the HBM and abrogated DARC binding [16]. A peptide based on this alanine substitution mutant (hbs-kara) did not bind to heparin. V3 loops containing an HBM bound with high affinity to heparin. The binding affinities of these peptides on a heparin-sepharose column were not correlated with their net charge, again suggesting structural specificity in their binding to sulfated polysaccharides.

We tested the specificity of the subdomain 1 HBM interaction with sulfated polysaccharides other than heparin by designing an ELISA in which hbs-wt peptide binding to heparin-coated wells could be detected. The same sulfated polysaccharides found to be inhibitory in the erythrocyte invasion assay and the RII region binding assay could competitively inhibit the hbs-wt peptide from binding heparin in the ELISA, with no inhibition from chondroitin sulfate C. This suggests that there is a structural interaction between the HBM of subdomain 1 and sulfated polysaccharides that is responsible for inhibition of merozoite invasion. The ELISA format is much faster than whole-cell and other binding assays and capable of being used as a high-throughput screen for inhibitors of merozoite infection. It may also detect leads for binding inhibitors of other DBLs and HIV.

We next compared whether the sulfated polysaccharide binding by the subdomain 1 HBM and V3 loops were structurally similar to that of RANTES. We developed an ELISA to detect RANTES binding to heparin and then measured competitive inhibition of that binding. Only V3 loop peptides containing an HBM from strain MN competed in a, but not other V3 loop peptides or the subdomain 1 peptide. This was not expected since RANTES binds to CCR5 and DARC while HIV-1 strain MN does not bind to CCR5, but to CXCR4.

It is possible that the HBMs of the DBP and V3 loops and mimic one or both binding properties of chemokines; to glycosaminoglycans (GAGs) or to sulfated tyrosines on chemokine receptors. We have shown the HBM in subdomain 1 can bind to sulfated polysaccharides, but DBP requires sulfation of tyrosine (Tyr 41) on DARC for binding [30], and the HBM may interact with this as well. Discrimination of SU between CCR5 and CXCR4 can be conferred by amino acid changes in the V3 loop [2,31-34]. The V3 loop is one of four SU domains that can contribute to heparan sulfate binding [6,35]. CCR5 and CXCR-4 have amino-terminal sequences that include tyrosine sulfation that are believed to be essential for HIV binding and may interact in part with positive residues in V3 HBMs. Here, the V3 loop of MN may indeed contribute to binding of sulfated tyrosines on CXCR-4 but have greater similarity with RANTES than does subdomain 1 for GAG binding.

We further explored whether the HBM in the DBL family might have a conserved GAG binding function by comparing sulfated polysaccharide inhibition of the PkDBP and the *P. knowlesi *beta and gamma proteins. Among these, only PkDBP binds to DARC while the receptors for the other two proteins are unknown. Only the PkDBP was inhibited by sulfated polysaccharides, though in our previous study, alanine substitution of the HBM abrogated erythrocyte binding of the beta and gamma proteins. This argues against the HBM serving a conserved GAG binding function in the DBL family or of nonspecifically binding to GAGs. When treated with heparitinase, erythrocytes retain their binding to PvDBP, also arguing against GAG binding as the function of the HBM in this protein. We did not digest other cell surface proteoglycans, and though a detection antibody for heparan sulfate showed loss of signal (data not shown), it is not possible to know if the digestion was complete enough to eliminate all interactions with the DBP. Other members of the DBL family have been shown to bind heparan sulfate. The *P. falciparum *erythrocyte membrane protein (PfEMP) family is expressed on the surface of parasitized erythrocytes, can mediate erythrocyte rosetting and cytoadherence and also contains Duffy binding like (DBL) domains with many HBMs [36]. Erythrocyte rosettes can be disrupted by digestion of heparan sulfate [36,37].

The HBMs of chemokines appear to exhibit multiple functions, allowing for cell surface binding and extracellular matrix sequestration as well as direct binding to sulfated tyrosines on chemokine receptors. This polyvalency could aid in the sometimes promiscuous binding between chemokines and chemokine receptors. The HIV SU, PkDBP and PvDBP have convergently evolved to bind to chemokine receptors and may use HBM or positively charged regions to mimic chemokine binding to GAG or sulfated tyrosines on the chemokine receptors. Sulfated polysaccharides may specifically block HBM regardless of their role in receptor binding, but appear to block members of the DBL with either known GAG or chemokine binding functions. The HBM in subdomain 1 of the DBP is required for DARC binding and has specific sulfated polysaccharide binding properties that recapitulates the inhibition of the merozoites invading erythrocytes and region II binding to DARC. This makes the PvDBP HBM an attractive target for inhibition since it is a conserved motif, and some of the same inhibitors may block HIV, though there are differences in heparin binding specificity of the PvDBP HBM and V3 loops as shown by competitive inhibition of RANTES binding to heparin. We have developed a high throughput screen for these inhibitors based on a ELISA with the PvDBP HBM. NMR structures of heparin binding to chemokines are available and future studies could better define the nature of PvDBP interaction with polyanionic inhibitors.

## Competing interests

The authors declare that they have no competing interests.

## Authors' contributions

MJB performed the investigations described in this study. MJB and RFG conceived of the study, and RFG participated in its design and coordination and edited the manuscript. Both authors read and approved the final manuscript.
